# *In-vitro* evaluation of the anti-cariogenic effect of a hybrid coating associated with encapsulated sodium fluoride and stannous chloride in nanoclays on enamel^*^

**DOI:** 10.1590/1678-7757-2021-0643

**Published:** 2022-05-02

**Authors:** Sávio José Cardoso BEZERRA, Ítallo Emídio Lira VIANA, Idalina Vieira AOKI, Simone DUARTE, Anderson Takeo HARA, Taís SCARAMUCCI

**Affiliations:** 1 Universidade de São Paulo Faculdade de Odontologia Departamento de Dentística São Paulo Brasil Universidade de São Paulo, Faculdade de Odontologia, Departamento de Dentística, São Paulo, Brasil.; 2 Universidade de São Paulo Escola Politécnica Departamento de Engenharia Química São Paulo Brasil Universidade de São Paulo, Escola Politécnica, Departamento de Engenharia Química, São Paulo, Brasil.; 3 Indiana University School of Dentistry, Operative Dentistry and Dental Public Health Department of Cariology Indianapolis United States Indiana University School of Dentistry, Operative Dentistry and Dental Public Health, Department of Cariology, Indianapolis, United States.

**Keywords:** Biofilm model, Caries, Fluoride, Stannous, Smart Coatings

## Abstract

**Objective:**

The aim of this study is to test, *in vitro*, the anti-cariogenic effect of experimental hybrid coatings, with nano clays of halloysite or bentonite, loaded with sodium fluoride or with a combination of sodium fluoride and stannous chloride, respectively.

**Methodology:**

The varnish Fluor Protector (1,000 ppm of F-) was used as positive control and no treatment was the negative control. Enamel specimens (5 mm × 5 mm) were obtained from bovine teeth. The specimens (n=10) had their surfaces divided into two halves (5 mm × 2.5 mm each), in which one half received one of the treatments (Hybrid; Hybrid + NaF; Hybrid + NaF + SnCl_2_; Hybrid + NaF Loaded; Hybrid + NaF + SnCl_2_ Loaded). The specimens were submitted to a cariogenic challenge using a biofilm model (S. mutans UA159, for 5 days). Enamel surfaces both under and adjacent to the treated area were analyzed for mineral loss and lesion depth, by transverse microradiography. The pH of the medium was measured twice a day, and the fluoride release was analyzed. Additional specimens were submitted to confocal analysis.

**Results:**

Data were statistically analyzed by two-way ANOVA followed by Tukey test (α=0.05). None of hybrid groups were able to reduce the lesion depth; the Hybrid + NaF group, however, was able to reduce mineral loss differing from the negative control (p=0.008). The groups showed no significant difference in the pH measurement and fluoride release. Confocal analysis confirmed that for all groups the biofilm growth was similar.

**Conclusion:**

None of the hybrid groups reduced lesion depth, but the Hybrid + NaF group was able to promote protection against mineral loss.

## Introduction

Fluoride therapy is one of the main strategies used for caries control. There is a wide range of fluoridated products available, for either at-home or professional use. The mode of action of fluoride against caries is post eruptive and local.^1^ When constantly present in dental biofilm, it can reduce demineralization and enhance remineralization.^2^ Monovalent fluoride compounds, such as sodium fluoride are the most common. Stannous fluoride, by contrast, is a polyvalent fluoride compound containing metal cation, which has also shown to protect against caries.^3,4^ Technological advances have significantly improved some of the drawbacks associated with the use of stannous fluoride, such as its low stability and tooth staining.^3^

The anti-cariogenic mechanism of Sn^2^relies on two properties inherent to this cation, including antimicrobial function and high affinity with the apatite surfaces. The antimicrobial action takes place through the inhibition of microbial enzymes involved in the transport and metabolism of glucose.^4^ This appears to be a stable activity due to stannous good oral substantivity, especially in the biofilm.^4,5^ Stannous can also interact with the tooth surfaces, forming a protective layer composed of a variety of compounds,^6^ which can also interfere with the biofilm architecture.^4^

The use of professionally applied fluoride products is one preventive strategy used around the world in private and public health settings. The biannual application of fluoride varnish has shown to promote a decrease in caries incidence over two years, especially on high-risk patients.^7,8^ The varnishes have the ability to adhere to tooth surfaces, prolonging the contact time between fluoride and enamel. After application of the varnish, saliva bathes the varnish and dissolves the fluoride salt, allowing fluoride ions to diffuse out of the varnish and become absorbed into fluoride reservoirs within oral soft tissues, plaque, and teeth.^9^ Although the mode of action of the fluoride varnish against caries is not fully understood, it is known that the bioavailability of fluoride is fundamental for controlling the progression of this condition.^9,10^

Another possible approach to protect the tooth against cariogenic challenges would be the use of a smart hybrid coating material, which contains a stock of active agents encapsulated in nano clays that are available under demand for preventing caries. A previous study showed that experimental hybrid coatings can chemically adhere to the dental surfaces after an alkaline treatment, causing dentin tubule occlusion.^11^ These coatings are known to have anti-staining properties, in addition to thermal, chemical, and biological resistance, which are characteristics attributed to their inorganic components. They also have flexibility, film-forming ability, and possibility of adhesion, which are features related to their organic components.^11^ The use of this hybrid coating appears to be promising for many dental applications and has not been fully explored. To our knowledge, there are no published studies testing the hybrid coating as a possible preventive approach against caries.

Despite the benefits, hybrid coatings present some shortcomings, such as the development of pores and defects when constantly challenged, mechanically and chemically. In view of this, the idea of including agents with a potential protective effect against caries in these coatings is worth considering. Special attention should be directed toward fluoride and the combination of fluoride and stannous, since they could potentially act on any defects, reducing demineralization and enhancing remineralization.^3,12^ For these agents to act on demand, they could be loaded into nano clays, from which F^-^ and Sn^2^ would be slowly released into the oral environment at the time of the cariogenic challenge. This release would not only promote minerals deposition on enamel surface, but also potentially act on enamel subsurface, similarly to the fluoride varnish.^13^ A previous study^14^ showed the feasibility of loading sodium fluoride and stannous chloride into nano clays of halloysite and bentonite, with posterior release of these ions on different mediums (acidic and neutral).

The aim of this study is to test, *in vitro*, the anti-cariogenic effect of experimental hybrid coatings with sodium fluoride or with the combination of sodium fluoride and stannous chloride, which were loaded into nano clays of halloysite and bentonite, respectively. A dynamic biofilm model was used. The null hypotheses were: 1) the experimental hybrid coatings would not differ from the negative control regarding enamel mineral loss and lesion depth assessed after the cariogenic challenge on the treated surface; 2) enamel mineral loss and lesion depth would not differ between the experimental hybrid coatings and the negative control on the surface adjacent to treatment.

## Methodology

### Study Design

**Figure 1:**
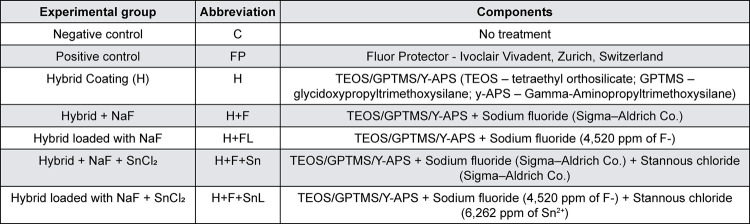
Experimental groups and components

**Figure 2:**
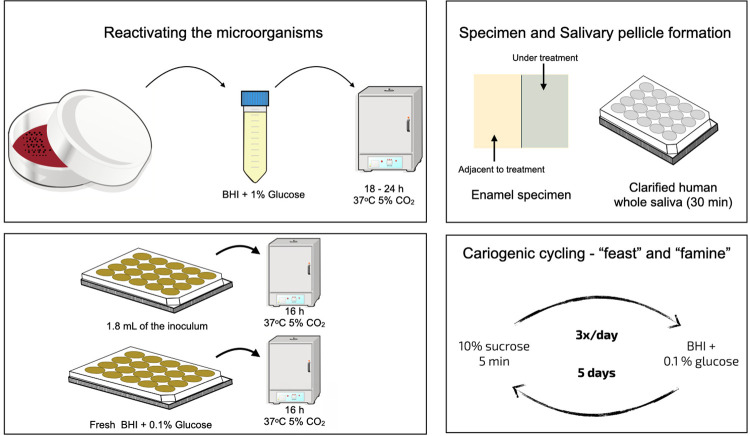
A flowgram of methodology

The study protocol was approved by the local ethics committee in research (#1406440799R001). This study followed a complete randomized design, with two experimental factors: treatments and specimen area (n=10). 1) Treatments, at 7 levels: C – negative control (no treatment); FP – positive control (Fluor Protector - Ivoclair Vivadent, Zurich, Switzerland); H – hybrid coating (TEOS/GPTMS/Y-APS (TEOS – tetraethyl orthosilicate; GPTMS – 3-glycidoxypropyltrimethoxysilane; y-APS – Gamma-Aminopropyltrimethoxysilane)); H+F – Hybrid + NaF (TEOS/GPTMS/Y-APS + Sodium fluoride (Sigma–Aldrich Co.)); H+FL – Hybrid loaded with NaF (TEOS/GPTMS/Y-APS + Sodium fluoride (4,520 ppm of F^-^)); H+F+Sn – Hybrid + NaF + SnCl_2_ (TEOS/GPTMS/Y-APS + sodium fluoride (Sigma–Aldrich Co.) + Stannous chloride (Sigma–Aldrich Co.)); H+F+SnL – Hybrid loaded with NaF + SnCl_2_ (TEOS/GPTMS/Y-APS + Sodium fluoride (4,520 ppm of F^-^) + Stannous chloride (6,262 ppm of Sn^2^)) (Figure 1). 2) Specimen’s area, at 2 levels: under-treatment and adjacent to treatment (Figure 2).

The treatments were tested using a dynamic cariogenic model with bovine enamel specimens (n=10). The enamel specimens (5 mm × 5 mm) were prepared and had their surface divided into two halves (5 mm × 2.5 mm each), one in which the treatment was applied (under-treatment), and the other that was adjacent to the treated area (adjacent to treatment). The specimens were submitted to the cariogenic challenge, using a biofilm model. The response variable was the mineral content change, measured by transverse microradiography and expressed by the total mineral loss and average lesion depth.

### Sample size calculation

A pilot study (not shown) was performed to determine the number of specimens per group. The sample size calculation was made on SigmaPlot 12.0. ANOVA Sample size was used, considering an effect size of -3784 for mineral loss and -119 for lesion depth, α=0.05 and a power of 0.80, obtaining a sample size of 5. Considering this and the previous studies that employed a similar methodology, we adopted n=10.

### Specimen Preparation

A total of 70 enamel slabs (5 mm × 5 mm) were sectioned from the crowns of the bovine incisors and was analyzed with a stereoscopic microscope to certify that they were free from demineralization, cracks, or any other defects. Bovine incisors were used instead human due to their chemical and physical similarities.^15,16^ After collection and during the preparation process, the teeth were stored in 0.1% thymol solution under refrigeration at 4ºC. The bottom and top (enamel) sides of the slabs were sequentially ground flat using silicon carbide grinding papers (#600, #1200, #2400 for 15 s, 25 s, and 30 s, respectively) (RotoPol 31 / RotoForce 4, Struers, Cleveland, Ohio, USA). The top side was serially polished up to a 4,000-grit grinding paper, followed by 1-μm diamond polishing suspension. Afterwards, they were sterilized in a steam autoclave, at 121ºC for 30 min followed by 10 min air-drying at sub-atmospheric pressure.

All the treated specimens were fixed in the lid of 24-well culture plates and were submitted to a cariogenic challenge, using a biofilm model^17^ for 5 days.

### Hybrid coating

The hybrid coating was prepared as previously described,^11,18^ contained a gamma-aminopropyltriethoxysilane (γ-APS), 3-glycidoxypropyltrimethoxysilane (GPTMS), and tetraethyl orthosilicate (TEOS). Briefly, TEOS and GPTMS were incorporated into a solution (alcohol plus water) and kept for 72 h under agitation. Afterwards, γ-APS was incorporated into the solution. The resulting solution was diluted with deionized water in the ratio of 1:3. For this study, a total of approximately 250 mL of hybrid coating was used. The total volume of the hybrid was divided into 5 experimental groups (50 mL/group). In the groups that had ions, they were incorporated following the protocol stablished in preliminary study.^14^ For groups with NaF (Sigma–Aldrich Co., St. Louis, MO, USA), a concentration of 0.1 g per 10 mL, resulting in 4,520 ppm of F^-^, was incorporated with 0.5 g of halloysite nano clay, under agitation, until solution became homogeneous. For groups with fluoride and stannous, NaF was added in same concentration and SnCl_2_ (Sigma–Aldrich Co.) in concentration of 0.1 g per 10 mL, resulting in a concentration of 6,261 ppm of Sn^2^ with 0.5 g of bentonite nano clay, under agitation, until solution became homogeneous. The different clays and ions concentration were established based on previous study.^14^

### Treatment application

For the treatment application, the 70 specimens were randomly allocated into 7 experimental groups (n=10), and the surfaces were divided into two equal halves, one submitted to treatments and other one just adjacent to treatment. The fluoride varnish (Fluor Protector) was applied in accordance with the manufacturer’s instructions, as a thin layer with the aid of a disposable applicator and with a 60 s wait for complete cure. For the hybrid coating groups, the protocol used was established in previous studies^11,14^ as follows: firstly, an alkaline surface treatment was performed with 0.05 M NaOH solution (pH of approximately 12.9) for 10 min, followed by rinsing with deionized water and drying. Then, the experimental hybrid solutions were applied with disposable applicator, in two layers. After each layer, the solution was allowed to dry for 4 min followed by application of heat source with a light curing device (Valo, Ultradent, South Jordan, UT, USA) for 60 s, at irradiance of 1,000 mW/cm^2^, with 1 mm apart, approximately, to complete the cure of each hybrid layer.

### Biofilm model

**Figure 3:**
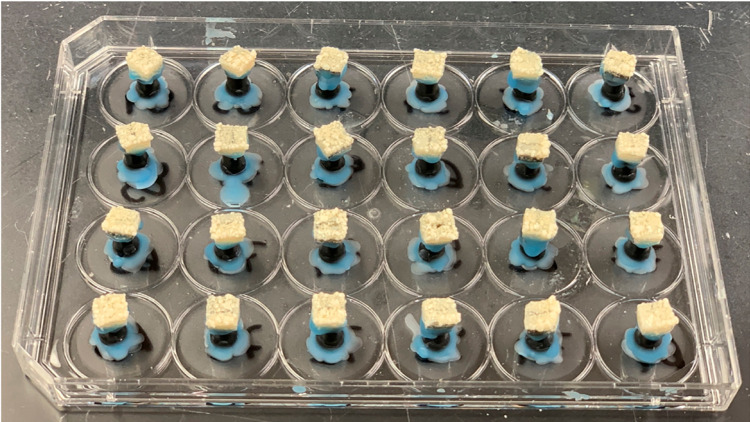
Image of enamel slabs positioned in cell plates cover

**Figure 4:**
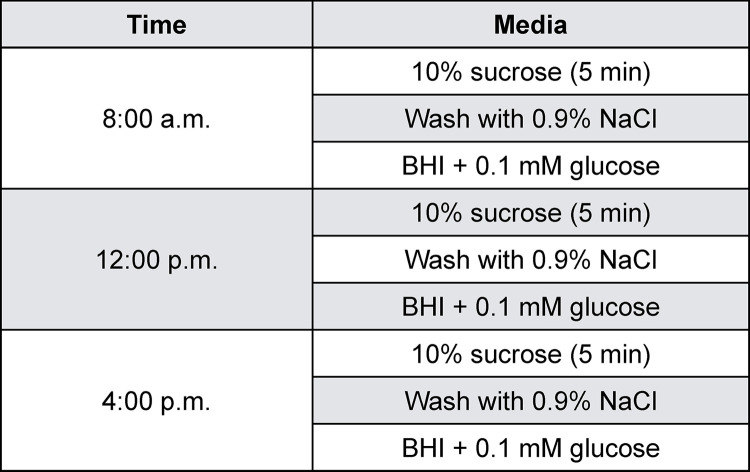
Cariogenic cycling

The cariogenic biofilm model used was based on a previous study,^17^ with modifications according to preliminary test results (data not published). *Streptococcus mutans* UA159 reference strain^19^ was used in the experiment. Depending on the experimental phase, the content of the media was 1% glucose, 1% sucrose or 0.1% glucose, as described below. *S. mutans* colonies were transferred to Brain Heart Infusion (BHI) supplemented with 1% glucose and were incubated for 18 h – 24 h at 37°C, 5% CO_2_ to reactivate the microorganisms. Slabs were first individually positioned in cell plates cover (Figure 3), then they were coated with human saliva, and a salivary pellicle was formed by being incubated in filter-sterilized clarified human whole saliva (IRB #1406440799R001) for 30 min, at 37ºC. After salivary pellicle formation, they were placed in 1.8 mL of the inoculum and were incubated at 37°C, 5% CO_2_ to allow bacterial adhesion on the acquired pellicle. Sixteen hours (previously standardized) after incubation the slabs were transferred to fresh BHI containing 0.1% glucose (salivary basal concentration) and incubated for an additional 16 h at 37°C, 5% CO_2_. All these procedures were performed according to Ccahuana-Vásquez and Cury^17^ (2010). At the beginning of second day, the biofilms on enamel slabs were transferred to fresh BHI containing 0.1 % glucose and were exposed to episodes of “feast” and “famine” comprised of 3 daily exposures to carbohydrate solutions: 10% sucrose (containing 1.23 mM Ca, 0.74 mM Pi and 0.023 μg F/mL, previously standardized) at predetermined times (08:00 a.m., 01:00 p.m., 06:00 p.m.) for 5 min. The frequency of exposure was chosen based on a pilot study to achieve an enamel lesion depth of approximately 138.85 µm (not published). This procedure was repeated in the next five days until the end of the experiment. After each sucrose exposure, the biofilms on enamel slabs were washed 2 times in 0.89% NaCl (Figure 2 and Figure 4). After the cariogenic challenge, the enamel slabs were rinsed 3 times in 0.89% NaCl solution and placed back into the culture media. The culture media was changed twice a day (in the beginning and at the end of cariogenic challenge), and the pH of culture media was determined twice a day, 2 h after the culture media was changed.

### Microradiography Analysis (TMR)

Specimens were sectioned with a hard tissue microtome (Silverstone-Taylor Hard Tissue Microtome, Series 1000 Deluxe; Silverstone-Taylor, SciFab, Lafayette, Colo., USA). One 100 μm section was obtained from each specimen, with both areas (treated and adjacent to treatment). Sections were mounted on X-ray-sensitive plates (Microchrome Technology Inc., San Jose, Calif., USA) and subjected to X-ray, along with an aluminum step wedge. Microradiographic images were analyzed with dedicated software (Inspektor TMR 2000, ver.1.25) with 87% set as a reference level for the average mineral content of sound enamel by volume. Two parameters were obtained for each specimen, the difference in between the two areas (under-treatment and adjacent treatment) was expressed as integrated mineral loss (ΔZ=vol%min × µm) and lesion depth (ΔL=µm). The investigator was blinded with respect to group allocation.

### Confocal Scanning Laser Microscopy (CSLM) Analysis

Additional specimens were used for the confocal analysis to determine the biofilm morphology, using a Leica TCS SP5 microscope (Leica Lasertechnik, Heidelberg, Germany). The biofilm on samples was stained with a live/dead viability kit (Molecular Probes; Invitrogen, Eugene, OR, USA). The green stains all cell populations (live and dead) and the red penetrates only through damaged cell membranes. Thus, when both fluorophores are used, live bacteria were stained green, and dead were stained red in CSLM images. The stain was prepared by diluting 1.5 μL of SYTO 9 and 1.5 μL of propidium iodide in 2.8 mL of sterile 0.89% NaCl solution.^20^ The plate containing the stains with the samples was incubated at room temperature, in the dark, for 15 min. After that, samples were washed three times in 0.89% NaCl solution and examined under a CSLM. The biofilm morphology was qualitatively analyzed.

### Fluoride Analysis

Aliquots of 1 ml of all media were collected twice a day, immediately after the culture media was changed. The aliquot was then mixed 1:1 with TISAB II (0.5 mL of sample + 0.5 mL of TISAB II) and analyzed for fluoride by comparison to a similarly prepared standard curve (1 mL standard + 1 mL TISAB II) using an ion-selective electrode (Orion Research, Boston, MA, USA). Fluoride data were calculated as µg F/mg (mean amount per specimen).

### Statistical Analysis

Data of Mineral Loss and Lesion Depth were statistically analyzed and were individually tested for normal distribution and homoscedasticity with Shapiro-Wilk and Brown Forsythe test, respectively. Considering that the data followed a normal distribution, a two-way repeated measurements ANOVA, followed by Tukey post-hoc test, was used for assessment. A 5% of level of significance was considered and the Sigma Plot (12.0) software was used for calculations.

## Results

**Figure 5:**
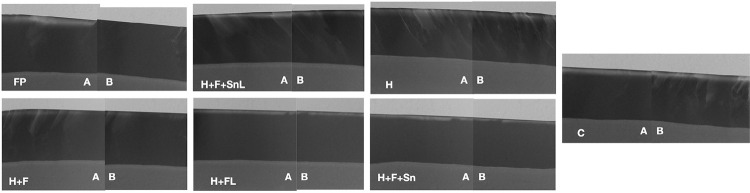
Representative TMR images of each treatment. A: surface adjacent to treatment; B: surface under treatment

The microradiography (Figure 5) results are shown in Table 1 (mineral loss) and Table 2 (lesion depth). For mineral loss, on the under-treatment surface, the Fluor Protector was the group that showed the lowest mineral loss, significantly differing from the negative control (p<0.001) and the other groups (p<0.001). The Hybrid + NaF group showed significant difference from negative control (p=0.008), but not from the groups with the loaded agents (p>0.005). The Hybrid + NaF + SnCl_2_ group, Hybrid group, and the groups with the loaded agents did not show a significant difference from the negative control (p>0.005). The surface adjacent to treatment did not show significant difference among groups (p>0.005). When the surfaces were compared, Fluor Protector, Hybrid + NaF, and the groups with loaded agents showed significant differences between surfaces (p<0.001), with a greater mineral loss on the surface adjacent to treatment.

**Table 1:** Mean and standard deviation (SD) of mineral loss (Vol%.μm], for both areas

	Under-Treatment		Adjacent to Treatment	
	Mean	SD	Mean	SD
Fluor Protector	^Aa^ 325.56	346.0	^Ab^ 3350.28	1811.4
Hybrid + NaF	^Ba^ 2226.44	603.9	^Ab^ 3655.00	781.4
Hybrid + NaF Loaed	^BCa^ 2987.78	495.8	^Ab^ 4036.50	1039.0
Hybrid + NaF + SnCl_2_ Loaed	^BCa^ 3225.56	923.0	^Ab^ 4151.00	1340.6
Hybrid + NaF + SnCl2	^Ca^ 3949.00	1663.7	^Aa^ 3238.00	1129.9
Hybrid	^Ca^ 3950.20	1280.3	^Aa^ 3772.00	1219.0
Control	^Ca^ 4110.00	1414.5	^Aa^ 3586.00	1221.7

Data analyzed by two-way repeated measured ANOVA. Different uppercase letters show statistical difference in a column by Tukey test. Different lowercase letters show statistical difference in a row by Tukey test

**Table 2:** Mean and SD of Lesion Depth (in μm), for both areas

	Under-Treatment		Adjacent to Treatment	
	Mean	SD	Mean	SD
Fluor Protector	^Aa^ 15.72	18.8	^Bb^ 120.58	42.4
Hybrid + NaF	^Ba^ 110.18	14.3	^ABb^ 151.73	27.5
Hybrid + NaF Loaed	^Ba^ 113.74	15.1	^Ba^ 123.62	26.5
Hybrid + NaF + SnCl2 Loaded	^Ba^ 127.14	42.0	^Aa^ 146.27	38.8
Hybrid + NaF + SnCl2	^Ba^ 153.32	50.5	^Aa^ 137.09	53.8
Hybrid	^Ba^ 129.66	30.5	^Aa^ 185.94	83.1
Control	^Ba^ 135.52	44.3	^Ba^ 114.53	22.1

Data analyzed by two-way repeated measured ANOVA. Different uppercase letters show statistical difference in a column by Tukey test. Different lowercase letters show statistical difference in a row by Tukey test

Regarding lesion depth, Fluor Protector was the group that showed the lowest lesion depth and the only one that differed significantly from the negative control (p<0.001), on the under-treatment surface. On the surface adjacent to treatment, Fluor Protector, Hybrid, and Negative Control groups showed statistically significant differences from the other groups (p<0.001), which did not differ from each other (p>0.005). Comparing surfaces, only Fluor Protector and Hybrid + NaF showed significant differences (p<0.001 and p=0.14, respectively), with greater lesion depth on the surface adjacent to treatment.

**Figure 6:**
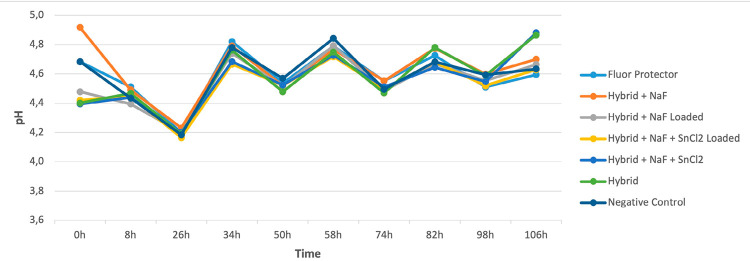
pH measurement of the medium during the cycling

**Figure 7:**
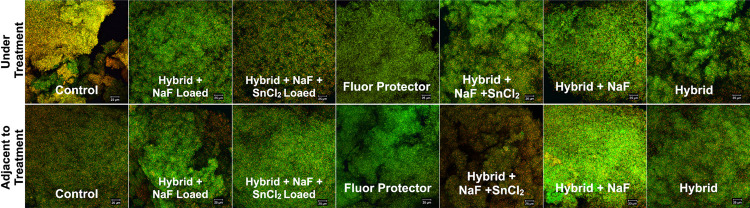
Images after staining with the Live/dead staining viability kit. Vital microorganism (green) and dead bacteria (red) are detectable at the enamel surface areas in the under-treatment and adjacent to treatment, for all experimental groups

**Figure 8:**
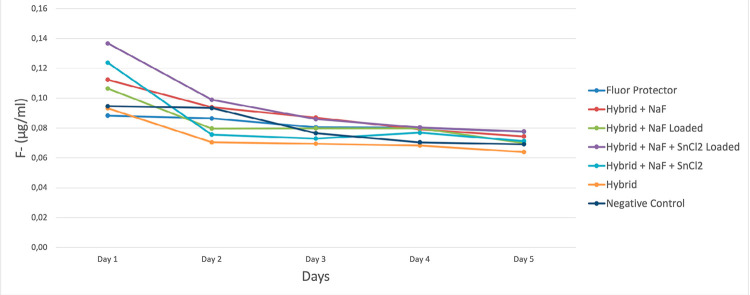
Fluoride release (µg/mL) over the cycling days

The range of pH analysis of the biofilm was from 4.4 to 4.9 over the five days of cycling, with no differences among the groups (Figure 6). According to the confocal analysis (Figure 7), the biofilm showed similar behavior for all groups. We observed that Negative control, Hybrid, and Hybrid + NaF + SnCl_2_ treatment resulted in a cluster-like architectural biofilm, as opposed to confluent biofilms that developed following the other treatment exposures. In the fluoride analysis (Figure 8), all the experimental groups had a similar amount of fluoride released on the medium at the end of the cycling.

## Discussion

The first null hypothesis of the study, which stated that the experimental hybrid coatings would not differ from the negative control as regarding mineral loss and lesion depth of the enamel assessed after the cariogenic challenge on the treatment surface, was rejected since the experimental hybrid coating with NaF was able to reduce the mineral loss of the enamel in more than 50% when compared to the negative control group. This could be attributed to the fluoride present in the hybrid coating, which was slowly released and was able to protect the surface against cariogenic acid. The Hybrid coating group, however, was not capable of reducing lesion depth significantly. The application of the hybrid coating solution on a polished enamel surface could have interfered with the mechanical retention of the coating during cycling, leading to its partial loss. Thus, the remaining material was not able to physically protect the surface against the challenges. Whereas, when the coating was loaded with fluoride, it served as a fluoride deposit, releasing this ion onto the lesion, optimizing remineralization.

This low retention of the hybrid coating to the enamel surface was also observed in a previous investigation,^14^ in which an initial demineralization was performed in the specimens to increase the retention of the coating. This initial demineralization simulates an already established lesion, which received the treatment with the coating to slow down its progression. To increase the hybrid adhesion, the authors suggests that a micro-retention with phosphoric acid, or with citric acid in high concentration may be created in future studies. On a previous study^14^ from our group – using a different model, with more aggressive acid and with more exposure to the challenge than the one used in this study – the hybrid coating was able to significantly protect the enamel and dentin surface against dental erosion. In that study the specimens had been previously eroded when the hybrid was applied, and this micromechanical retention could have increased the adhesion of the coating.

A different behavior of the hybrid coating was expected in these different models, as it is known that dental caries and dental erosion are distinct processes. Dental erosion results in a demineralized surface, with the acid also affecting a near-surface layer; considered mainly a surface phenomenon. By contrast, dental caries in its initial process is a subsurface phenomenon, in which the destructive effects occur on the surface, but mostly within the subsurface region.^21^

The fluoride varnish (Fluor Protector) was the only group able to protect the under-treatment area against the cariogenic challenges, by reducing lesion depth and mineral loss. Fluor Protector (Ivoclair Vivadent, Zurich, Switzerland) is a regular varnish that contains fluoride (1% ppm of F^-^) and difluorosilane on its composition. The regular fluoride varnish may act by preventing biofilm formation and promoting a slow fluoride release, inhibiting demineralization and promoting enamel remineralization.^22^ In this sense, we believe that the fluoride varnish was able to adhere to the enamel surface, inhibiting the formation of lesions.

The second null hypothesis of this study was accepted since none of the groups were able to significantly protect the area adjacent to treatment. Although the fluoride varnish and the Hybrid + NaF loaded showed significantly lower lesion depth in relation to the other groups, they did not differ from negative control. When the areas were compared, the varnish and Hybrid + NaF groups were able to promote a smaller lesion depth on the under-treatment surface. Furthermore, these groups and the loaded groups (Hybrid + NaF Loaded and Hybrid + NaF + SnCl_2_ Loaded), reduced the mineral loss of the same area when compared with area adjacent to treatment. We believe that this slightly better protection was due to the action of the ions that were released by the clays presented on the remnants of coating.

A possible antimicrobial/antibiofilm effect of stannous was expected,^4^ considering the ability of stannous in changing *S. Mutans* growth and metabolism, as well as inhibiting bacterial adhesion, plaque formation, and acid production, potentially retarding biofilm development and altering its architecture.^4^ This, however, was not possible to observe in our study, possibly due to the detachment of the material at the beginning of the challenge. According to the pH analysis, none of the experimental groups was able to inhibit the decrease in the biofilm pH; and, on the fluoride release analysis, all groups showed similar amount of fluoride during all the experiment. The confocal analysis showed that the biofilm growth was similar in all experimental groups, confirming the demineralization by the acids derived from the microorganisms present in the biofilm. The confocal analysis coincides with pH, as no treatment was able to reduce acid production or inhibit bacterial adhesion.

Considering the lack of effect on biofilm pH control and amount of fluoride released observed in the Fluor Protector group, we believe that its protective quality is attributed to the mechanical barrier provided by the varnish. Fluor Protector was able to adhere to the enamel surface, promoting a mechanical barrier and preventing direct contact of acids on the surface. These controversial results indicate that mechanical property of the fluoride varnish was potentially greater than chemical-mechanical action. Fluor Protector is a fluoride varnish that has been used for many years in the USA and Europe,^23^ and its effect against caries demineralization has already been shown in many studies.^24-26^ This varnish has sodium fluoride (1,000 ppm F) and silane in its formulation. The anti-cariogenic action of the fluoride in this varnish is basically related to an incorporation of fluoride into the crystalline lattice of enamel and its interaction with saliva, forming calcium fluoride (CaF_2_) compounds on the enamel.^23^

Despite the good protection results observed in the TMR analysis and considering the results of the fluoride analysis, we believe that a significant fluoride effect was not present in this study. Some studies observed that remineralization with Fluor Protector was greater than other materials.^27-29^Most of these studies, however, used a chemical caries model; whereas our study used a more aggressive biofilm caries model. This model was chosen to simulate the clinical scenario in a more significant manner. There is a wide variety of cariogenic biofilm models in the literature, showing a great variation on the type of microorganisms,^30^ cariogenic challenge frequency,^17^ and duration.^31^ After a pilot study (not shown), the protocol with 3 challenge/day for 5 days was the one that promoted a caries lesion for detection by the microradiography technique, and that would not promote enamel surface loss. Moreover, the model chosen was biased towards demineralization than remineralization. In view of this, it can be argued that, in a remineralization-bias model, a better action of Fluor Protector and of the Hybrid + NaF would be observed. Another limitation of the study, regarding the methodology, could be the autoclaving process of the samples. Even if there are some detrimental effects, it was a step needed to ensure the quality of the work and it was done equally to all experimental groups.

In a previous study,^25^ an increased fluoride retention on enamel was observed when combining the applications of fluoridated varnish and mouth rinse, thus, this could be a promising approach to be tested in the future. As assumed in another study,^32^ the amount of fluoride release from fluoride products would depend on the differences in solubility and release of fluoride among these products. Fluoride release from fluoride varnishes was investigated on different environments, and the authors observed that it varies considerably among products, being also dependent on the dissolution medium.^33^ Although Fluor Protector promoted better enamel protection against demineralization, the results of our study showed that Hybrid + NaF coating was the best treatment when compared to the other hybrid treatments. It has the potential to be used in future studies as a protective product against demineralization, given that its retention to the enamel surface is improved.

Surprisingly, the groups with nano containers (halloysite and bentonite) loaded with fluoride and stannous ions did not show significant effect under the circumstances of this study, when compared to the control group. It is already known that the addition of nano containers to polymeric films may improve their mechanical resistance, without compromising the performance of the material.^34^ The addition of halloysite clay nanotubes loaded with chlorhexidine into bonding agents did not change their viscosities.^35^ In this study, however, an increased viscosity of the hybrid coating was visually observed, which lead us to hypothesize that the amount of nano clays added was excessive, to a point that its adhesion to the enamel substrate was further jeopardized. The more viscous the hybrid solution, the harder it would be for it to penetrate irregularities of the enamel, allowing the coating to be more easily detached from the enamel surface. Thus, the ions had short time to interact with the enamel surfaces, showing lower protective effect.

Additionally, the hybrid coating was a dense network and may have acted as a barrier, not properly allowing the releasing of ions, even for non-encapsulated agents. It is known that, after the cure of the hybrid solution, a crosslinking occurs resulting in chemically stable bonds with the tooth surface; allowing the hybrid coating to act as a mechanical barrier against the acid challenge.^14^ As described on previous study,^14^ the material needs an adequate cure to achieve good properties, which should be performed at high temperatures.^36^ We believe that the cure performed in this study may not have been enough to promote satisfactory adhesion of the material to the surface. Therefore, more studies are necessary to improve the cure of the hybrid coating.

Moreover, further studies need to be conducted to enhance the adhesion of this material to dental substrates, considering that this treatment would be indicated for professional use, especially in areas of the teeth that already had some demineralization and irregularities. A less aggressive model should be used in future studies to test the hybrid coatings, which might give more opportunities to the ions to be released slowly and be able to protect the enamel surface.

## Conclusion

Fluor Protector showed greater protection against cariogenic challenge. None of the hybrid coatings treatments reduced lesion depth; nevertheless, the Hybrid with NaF prevented enamel mineral loss. Future studies aiming to improve the retention of the hybrid coatings on sound enamel are needed, in order to optimize its effect.
